# Author Correction: Genome sequencing and population genomic analyses provide insights into the adaptive landscape of silver birch

**DOI:** 10.1038/s41588-019-0442-7

**Published:** 2019-06-13

**Authors:** Jarkko Salojärvi, Olli-Pekka Smolander, Kaisa Nieminen, Sitaram Rajaraman, Omid Safronov, Pezhman Safdari, Airi Lamminmäki, Juha Immanen, Tianying Lan, Jaakko Tanskanen, Pasi Rastas, Ali Amiryousefi, Balamuralikrishna Jayaprakash, Juhana I Kammonen, Risto Hagqvist, Gugan Eswaran, Viivi Helena Ahonen, Juan Alonso Serra, Fred O Asiegbu, Juan de Dios Barajas-Lopez, Daniel Blande, Olga Blokhina, Tiina Blomster, Suvi Broholm, Mikael Brosché, Fuqiang Cui, Chris Dardick, Sanna E Ehonen, Paula Elomaa, Sacha Escamez, Kurt V Fagerstedt, Hiroaki Fujii, Adrien Gauthier, Peter J Gollan, Pauliina Halimaa, Pekka I Heino, Kristiina Himanen, Courtney Hollender, Saijaliisa Kangasjärvi, Leila Kauppinen, Colin T Kelleher, Sari Kontunen-Soppela, J Patrik Koskinen, Andriy Kovalchuk, Sirpa O Kärenlampi, Anna K Kärkönen, Kean-Jin Lim, Johanna Leppälä, Lee Macpherson, Juha Mikola, Katriina Mouhu, Ari Pekka Mähönen, Ülo Niinemets, Elina Oksanen, Kirk Overmyer, E Tapio Palva, Leila Pazouki, Ville Pennanen, Tuula Puhakainen, Péter Poczai, Boy J H M Possen, Matleena Punkkinen, Moona M Rahikainen, Matti Rousi, Raili Ruonala, Christiaan van der Schoot, Alexey Shapiguzov, Maija Sierla, Timo P Sipilä, Suvi Sutela, Teemu H Teeri, Arja I Tervahauta, Aleksia Vaattovaara, Jorma Vahala, Lidia Vetchinnikova, Annikki Welling, Michael Wrzaczek, Enjun Xu, Lars G Paulin, Alan H Schulman, Martin Lascoux, Victor A Albert, Petri Auvinen, Ykä Helariutta, Jaakko Kangasjärvi

**Affiliations:** 1 Division of Plant Biology, Department of Biosciences, University of Helsinki, Helsinki, Finland; 2 Viikki Plant Science Centre, University of Helsinki, Helsinki, Finland; 3 Institute of Biotechnology, University of Helsinki, Helsinki, Finland; 4 Green Technology, Natural Resources Institute Finland (Luke), Helsinki, Finland; 5 Department of Biological Sciences, University at Buffalo, Buffalo, New York, USA; 6 Department of Zoology, University of Cambridge, Cambridge, UK; 7 Green Technology, Natural Resources Institute Finland (Luke), Haapastensyrjä, Läyliäinen Finland; 8 Department of Environmental and Biological Sciences, University of Eastern Finland, Kuopio, Finland; 9 Department of Forest Sciences, University of Helsinki, Helsinki, Finland; 10 Molecular Plant Biology, Department of Biochemistry, University of Turku, Turku, Finland; 11 Department of Agricultural Sciences, University of Helsinki, Helsinki, Finland; 12 Institute of Technology, University of Tartu, Tartu, Estonia; 13 Appalachian Fruit Research Station, Agricultural Research Service, United States Department of Agriculture, Kearnysville, West Virginia USA; 14 Umeå Plant Science Centre, Department of Plant Physiology, Umeå University, Umeå, Sweden; 15 Division of Genetics, Department of Biosciences, University of Helsinki, Helsinki, Finland; 16 Department of Biosciences, University of Helsinki, Helsinki, Finland; 17 DBN Plant Molecular Laboratory, National Botanic Gardens of Ireland, Dublin, Ireland; 18 Department of Environmental and Biological Sciences, University of Eastern Finland, Joensuu, Finland; 19 Department of Haemato-oncology, King’s College London, London, UK; 20 Department of Environmental Sciences, University of Helsinki, Helsinki, Finland; 21 Institute of Agricultural and Environmental Sciences, Estonian University of Life Sciences, Tartu, Estonia; 22 Finnish Museum of Natural History (Botany), University of Helsinki, Helsinki, Finland; 23 Management and Production of Renewable Resources, Natural Resources Institute Finland (Luke), Helsinki, Finland; 24 Department of Plant Sciences, Norwegian University of Life Sciences, Ås, Norway; 25 Institute of Plant Physiology, Russian Academy of Sciences, Moscow, Russia; 26 Genetics and Physiology Unit, University of Oulu, Oulu, Finland; 27 Forest Research Institute Karelian Research Centre Russian Academy of Sciences, Petrozavodsk, Russia; 28 Department of Ecology and Genetics, Evolutionary Biology Center and Science for Life Laboratory, Uppsala University, Uppsala, Sweden; 29 Sainsbury Laboratory, University of Cambridge, Cambridge, UK; 30 Present Address: Ecological Genetics Research Unit, Department of Biosciences, University of Helsinki, Helsinki, Finland; 31 Present Address: National Institute of Health and Welfare (THL), Kuopio, Finland; 32 Present Address: Finnish Institute of Occupational Health, Work Environment Laboratories, Kuopio, Finland; 33 Present Address: Institute of Biotechnology, University of Helsinki, Helsinki, Finland, and Division of Plant Biology, Department of Biosciences, University of Helsinki, Helsinki, Finland; 34 Present Address: School of Forest Biotechnology, Zhejiang Agriculture and Forestry University, Hangzhou, China; 35 Present Address: Unité AGRI′TERR, UniLaSalle, Campus de Rouen, Mont-Saint-Aignan, France; 36 Present Address: Blueprint Genetics, Helsinki, Finland; 37 Present Address: Sainsbury Laboratory, University of Cambridge, Cambridge, UK; 38 Present Address: Institute of Botany, The Chinese Academy of Sciences, Beijing, China; 39 Present Address: Green Technology, Natural Resources Institute Finland (Luke), Helsinki, Finland; 40 Present Address: Agricultural and Food Science/Scientific Agricultural Society of Finland, Lemu, Finland; 41 Present Address: Royal Haskoning DHV, Maastricht Airport, Beek, the Netherlands; 42 Present Address: Chemistry and Toxicology Research Unit, Finnish Food Safety Authority Evira, Helsinki, Finland

Correction to: *Nature Genetics* 10.1038/ng.3862, published online 8 May 2017.

In the version of this article initially published, there was a mistake in the calculation of the nucleotide mutation rate: 1 × 10^−9^ mutations per site per generation was used, whereas the regression formula given by Lynch (M. Lynch, *Trends Genet*. **26**, 345–352; 2010), which was used in the published article, in fact provides a rate of 9.5 × 10^−9^ mutations per site per generation. This error affects the interpretation of population-size changes over time and their possible correspondence with known geological events, as shown in the original Fig. 4 and supporting discussion in the text, as well as details in the Supplementary Note. Neither the data themselves nor any other results are affected.

Herein, the range of mutation rates and generation times is revised for silver birch to better reflect uncertainties in both parameters. These uncertainties are large in long-lived forest tree species, but the issue at hand is not specific to them. Even in a species as well studied as humans, there is still substantial uncertainty regarding the mutation rate, with a twofold difference between indirect estimates at ~10^–9^ per base pair per year according to divergence, and direct estimates (obtained from pedigrees), in which estimates of approximately 0.5 × 10^–9^ have been obtained (P. Moorjani, Z. Gao and M. Przeworski, *PLoS Biol*. **14**, e2000744; 2016). The latter rate was used in the article that introduced the stairway plot method (X. Liu and Y.-X. Fu, *Nat. Genet*. **47**, 555–559; 2015). Regarding generation times, in natural stands, birch begins flowering around the age of 10 years. At the other extreme, the maximum age of a birch tree can be up to 120–150 years. An intermediate (average) value between age at maturity and maximum observed life span was used to estimate generation time in the tropical palm *Euterpe globosa* (L. Van Valen, *Biotropica* **7**, 259–269; 1975, and R. J. Petit and A. Hampe, *Annu. Rev. Ecol. Evol. Syst*. **37**, 187–214; 2006). Following a similar strategy would give a maximum generation time of 80 years for birch. The generation-time range was therefore revised in the calculations to the values 10, 40 and 80 years.

Because there are no estimates of the mutation rate available for birch, estimates from other plant species must be used as a proxy. For example, for *Populus trichocarpa*, a mutation rate was calculated on the basis of *Arabidopsis thaliana* mutation rates and fossil evidence (G. A. Tuskan et al., *Science* **313**, 1596–1604; 2006). Studies of mutation-accumulation lines in *A. thaliana* have resulted in estimates of 7.0 × 10^−9^ to 7.4 × 10^−9^ mutations per site per generation (S. Ossowski et al., *Science* **327**, 92–94; 2009, and S. Yang et al., *Nature* **523**, 463–467; 2015). However, estimates of *A. thaliana* generation times in nature vary between 1.3 years (M. Falahati-Anbaran, S. Lundemo and H. K. Stenøien, *New Phytol*. **202**, 1043–1054 (2014) and 4 years (S. Lundemo, M. Falahati-Anbaran and H. K. Stenøien, *Mol. Ecol*. **18**, 2798–2811; 2009), depending on the effects of seed banks. Altogether, this would result in a potential range of 1.75 × 10^−9^ to 6.7 × 10^−9^ mutations per site per year for *A. thaliana*. The lower bound agrees with a recent estimate from natural populations, wherein rates varied between 2 × 10^−9^ and 5 × 10^−9^ mutations per site per year (M. Exposito-Alonso et al., *PLoS Genet*. **14**, e1007155; 2018). Dividing by six, as in Tuskan et al. (G. A. Tuskan et al., *Science* **313**, 1596–1604; 2006) to better agree with fossil evidence, and multiplying by the generation time of birch (by using a range of 10, 40 and 80 years) would result in approximate lower bounds at 2.9 × 10^−9^, 1.2 × 10^–8^ and 2.3 × 10^–8^, and upper bounds at 1.2 × 10^–8^, 4.5 × 10^–8^ and 8.9 × 10^–8^ mutations per site per generation, respectively.

Alternatively, mutation-rate estimates from other woody perennials can be used. In peach (*Prunus persica*), another woody tree species, parent-progeny sequencing has yielded a mutation rate of 7.77 × 10^−9^ point mutations per site per generation (Z. Xie et al., *Proc. Biol. Sci*. **283**, 20161016; 2016), which is similar to the estimates for *A. thaliana* from mutation-accumulation lines (S. Ossowski et al., *Science* **327**, 92–94; 2009, and S. Yang et al., *Nature* **523**, 463–467; 2015), thus suggesting that mutation rate may not be dependent on generation time, possibly because of purifying background selection (M. Exposito-Alonso et al., *PLoS Genet*. **14**, e1007155; 2018). Thus, one estimate of birch mutation rate can be obtained by simply assuming that the mutation rate per generation is similar across all woody tree species.

To account for the large uncertainty in mutation-rate estimates discussed above, Fig. 4 has been revised to incorporate a range of values. The mutation rate is first plotted by the rate estimated for peach, 7.77 × 10^−9^, and then the respective lower and upper bounds for each generation time, 2.9 × 10^−9^, 1.2 × 10^–8^, 2.3 × 10^–8^, 1.2 × 10^–8^, 4.5 × 10^–8^ and 8.9 × 10^–8^ mutations per site per generation, respectively. The curve of changes in effective population size (*N*_e_) is not altered, but the absolute dates for the *N*_e_ bottlenecks differ according to the assumed mutation rate and generation time. As such, the oldest bottleneck observed may be as young as ~2.6 million years ago (Mya) or as old as 34 Mya, instead of at the Cretaceous–Paleogene boundary, as reported in the original figure. Such a difference of greater than an order of magnitude highlights the problems inherent in all attempts to provide calendar dates for population genomic features, especially in long-lived perennials in which there is vast uncertainty in generation times and mutation rates.

Images of the original and corrected figure panels are shown in the correction notice.

**Fig. 4:**
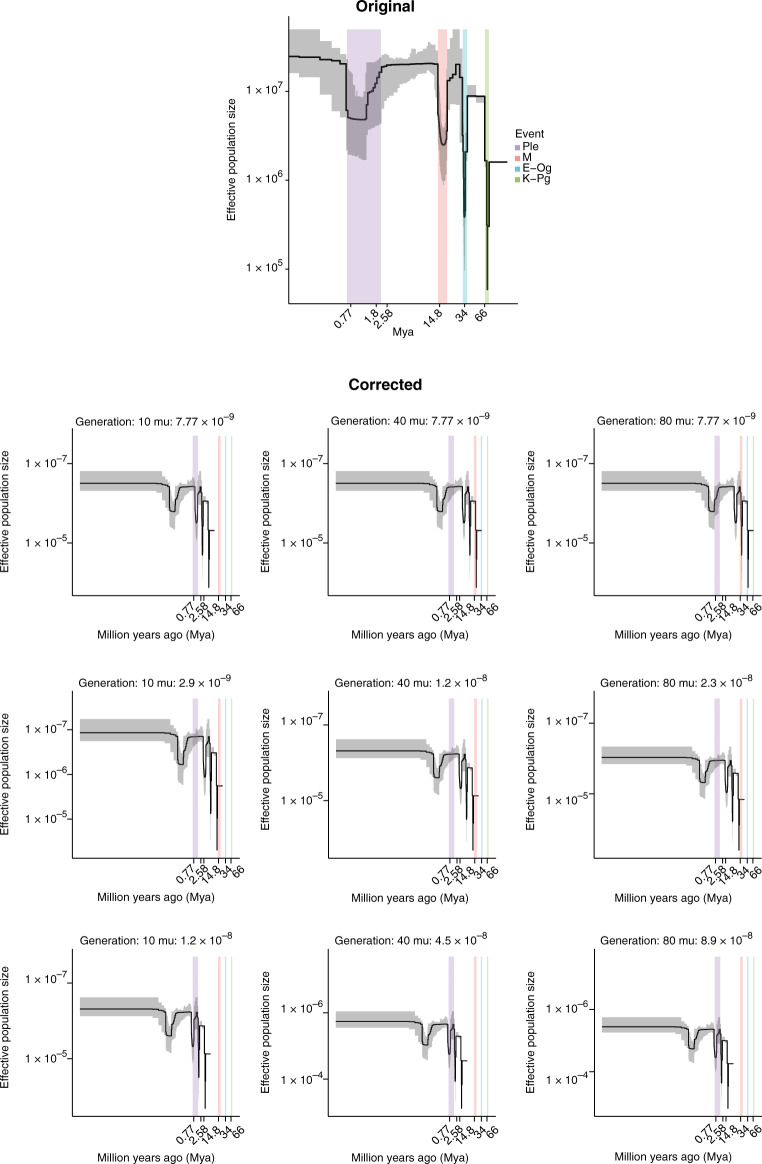
Historical effective population size for silver birch. Top, original figure with incorrect mutation rate; bottom, corrected figure. Varying mutation rates and generation times (described herein) were used to estimate historical population dynamics. The stairway plot shows that the *B. pendula* population has undergone bottlenecks that may be associated with some of the four known periods of major climate upheaval: the Cretaceous–Paleogene (K–Pg; green) the Eocene–Oligocene (E–Og; blue), the mid-Miocene (M; red) and the Pleistocene (Ple; purple). Data are median estimates from 200 bootstrap replicates (black lines) and 95% confidence intervals (shading). Tick marks along the *x* axis show estimates for the Matuyama–Brunhes (0.77 Mya), Calabrian (1.8 Mya) and Gelasian (2.58 Mya) borders, as well as the mid-Miocene disruption (14.5–14.8 Mya) and the E–Og (34 Mya) and K–Pg (66 Mya) events. As can be seen, no set of generation-time and mutation-rate assumptions yields an *N*_e_ dip near the Cretaceous–Paleogene boundary.

